# Practical Guide to Percutaneous Biopsy with Contrast-Enhanced Mammography: From Planning to Post-Procedural Management

**DOI:** 10.3390/diagnostics16162603

**Published:** 2026-08-17

**Authors:** Luciano Mariano, Fatemeh Darvizeh, Luca Nicosia, Alice Bonanomi, Sonia Santicchia, Isabella Castiglioni, Lorenza Meneghetti, Deborah Fazzini, Enrico Cassano

**Affiliations:** 1 Division of Breast Radiology, Department of Medical Imaging and Radiation Sciences, IEO European Institute of Oncology, IRCCS, Via Ripamonti 435, 20141 Milan, Italy; luciano.mariano@ieo.it (L.M.); alice.bonanomi@ieo.it (A.B.); sonia.santicchia@ieo.it (S.S.); lorenza.meneghetti@ieo.it (L.M.); enrico.cassano@ieo.it (E.C.); 2 Department of Diagnostic Imaging and Stereotactic Radiosurgery, Centro Diagnostico Italiano (CDI), Via Simone Saint Bon 20, 20147 Milan, Italy; isabella.castiglioni@cdi.it (I.C.); deborah.fazzini@cdi.it (D.F.); 3 Department of Physics “Giuseppe Occhialini”, University of Milan-Bicocca, Piazza della Scienza 3, 20126 Milan, Italy

**Keywords:** contrast-enhanced mammography, CEM-guided biopsy, MRI-guided biopsy, vacuum-assisted biopsy, breast cancer

## Abstract

Contrast-enhanced mammography-guided biopsy (CEM-guided biopsy) is an emerging minimally invasive technique for the diagnosis of enhancing breast lesions visible only after contrast administration. Traditionally, these lesions required magnetic resonance imaging (MRI)-guided biopsy, which may be limited by high costs, reduced accessibility, prolonged procedure times, and patient-related contraindications. This review aims to provide a practical and updated overview of CEM-guided biopsy, focusing on procedural workflow, technical execution, patient preparation, and complication management in daily clinical practice. A narrative review of the current literature on CEM-guided biopsy was performed, focusing on technical feasibility, procedural protocols, patient management, diagnostic performance, and reported complications. This review integrates evidence from recent clinical studies and practical considerations derived from interventional breast imaging practice. Early clinical experiences demonstrate high technical success rates for CEM-guided biopsy, with procedure times generally shorter than those reported for MRI-guided biopsy. However, formal measures of diagnostic accuracy have not been consistently reported across the available studies. Key procedural aspects include pre-procedural imaging evaluation, contrast administration timing, lesion targeting on recombined images, patient positioning, vacuum-assisted biopsy techniques, and post-procedural care. Reported complication rates are low and comparable to those of conventional stereotactic biopsy procedures. CEM-guided biopsy represents a promising alternative to MRI-guided breast biopsy, offering a faster, more accessible, and well-tolerated approach for sampling enhancing-only breast lesions. Although current evidence remains limited and heterogeneous, this technique may facilitate wider implementation of contrast-enhanced image-guided breast interventions. Standardized protocols and larger multicenter studies are still needed to validate its broader clinical role.

## 1. Introduction

In recent years, advances in Breast Cancer (BC) screening have focused on improving malignancy detection, particularly in patients with dense breasts [1]. Several studies demonstrate the benefits of integrating Magnetic Resonance Imaging (MRI) into personalized screening protocols for this patient cohort, reporting detection rates of up to 16.5 cancers per 1000 women and an increased identification of “enhancing-only lesions” [2,3]. These lesions are visible exclusively after contrast media administration, remaining undetectable with conventional breast imaging techniques such as Mammography or Ultrasound (US). They may account for up to 10% of all breast lesions and are associated with cancer in up to 50% of cases [4].

Until the Food and Drug Administration approved Contrast-Enhanced Mammography (CEM) in 2021, MRI was the only imaging modality to guide biopsy of these lesions. However, several limitations such as high costs, limited availability, procedural complexity, and contraindications specific to patients with claustrophobia or metal implants [5,6] restrict its use.

In this scenario, Contrast-Enhanced Mammography-guided Biopsy (CEM-guided biopsy) emerges as an innovative and promising technique for the percutaneous diagnosis of breast lesions, offering a more accessible, cost-effective, and generally better-tolerated alternative [7,8]. The combination of morphofunctional lesion visualization, increased patient comfort, and improved position flexibility makes it an effective solution for overcoming resource constraints and optimizing clinical workflows [8,9].

Studies on CEM-guided biopsy are still relatively few and involve limited patient cohorts; however, initial results demonstrate high technical success rates and substantially shorter procedure times than those reported for MRI-guided biopsy. Alcantara et al. reported a technical success rate of 95% and an average procedure duration of 15 min, compared with 35–41 min for MRI-guided biopsies [9]. Similar success rates have been reported in other investigations, with procedures lasting approximately 16–17 min with an acceptable radiation dose comparable to that of standard stereotactic techniques [10,11].

In recent years, several studies have explored the feasibility, diagnostic performance, and clinical implementation of CEM-guided biopsy. Initial experiences have consistently demonstrated high technical success rates, generally exceeding 90%, with procedure times significantly shorter than MRI-guided biopsy. Moreover, reported complication rates are low and comparable to those of standard vacuum-assisted biopsy techniques [9,10,11].

Subsequent single-center studies and early multicenter experiences have confirmed these findings, highlighting the reliability of CEM-guided biopsy in sampling enhancing-only lesions and its potential role as an alternative to MRI-guided interventions [4,10,11]. However, the available literature remains heterogeneous in terms of patient selection, technical protocols, and reported outcomes, reflecting the evolving nature of this technique. The principal published clinical experiences with CEM-guided biopsy are summarized in Table 1. Across the available studies, technical success has generally been high; however, differences in patient selection, lesion characteristics, study design, procedural protocols, and definitions of technical success limit direct comparisons. Malignancy rates also vary substantially, reflecting differences in referral populations and biopsy indications. Moreover, formal measures of diagnostic accuracy have not been consistently reported, as most studies primarily evaluated technical feasibility and early procedural outcomes rather than long-term diagnostic performance.

CEM-guided biopsy and MRI-guided biopsy should be considered complementary rather than interchangeable techniques. Although initial studies of CEM-guided biopsy have demonstrated high technical success rates, formal measures of diagnostic accuracy and direct head-to-head comparisons with MRI-guided biopsy have not been consistently reported. Consequently, the available evidence does not establish the diagnostic superiority of either technique.

CEM-guided biopsy generally offers shorter procedure times, with reported durations of approximately 14–17 min, compared with approximately 35–41 min for MRI-guided biopsy [4,5,9,10,11]. It may also be better tolerated because it can be performed in the upright, lateral decubitus, or prone position and avoids the confined MRI environment, making it potentially advantageous for patients with claustrophobia or MRI-related contraindications [6,7,8,12,13]. Furthermore, its implementation on suitably equipped mammography systems may improve accessibility, simplify scheduling, and reduce resource use compared with MRI-guided interventions [7,8].

Nevertheless, CEM-guided biopsy has specific limitations. It requires iodinated contrast medium administration and exposes the breast to ionizing radiation through repeated dual-energy acquisitions. Available initial studies suggest that radiation exposure is within the range reported for stereotactic breast biopsy procedures, although dose data remain limited and heterogeneous [10,11]. Technical success also depends on adequate lesion enhancement, inclusion of the target within the compression field, lesion depth, breast thickness, and maintenance of lesion conspicuity throughout targeting and sampling. Faint or rapidly fading enhancement, very posterior lesions, lesions close to the chest wall or axilla, and targets that cannot be adequately included within the biopsy window may make CEM guidance technically challenging or unfeasible [9,10,11,12,13].

MRI-guided biopsy therefore remains preferable when a suspicious lesion is visible only on MRI and cannot be reproduced on pre-procedural or procedural CEM, when the target cannot be adequately accessed with mammographic compression, or when iodinated contrast medium administration is contraindicated and MRI can be safely performed [6,7,8]. MRI guidance also avoids ionizing radiation, which may be particularly relevant for younger or high-risk patients undergoing repeated imaging. Accordingly, the choice of biopsy guidance should be individualized according to lesion visibility and location, patient-related contraindications, local equipment and expertise, procedural availability, and patient preference.

The following educational review explores the technical and clinical applications of CEM-guided biopsy, providing a practical overview for physicians approaching this technique. Key aspects include pre-procedural imaging assessment, patient preparation, patient positioning, biopsy technique and post-procedural management. Despite these encouraging results, the current body of evidence remains limited, and standardized protocols and large-scale comparative studies are not yet fully established.

In particular, CEM-guided biopsy presents unique technical and procedural characteristics compared to conventional stereotactic or MRI-guided approaches, including contrast timing, dual-energy acquisition, and lesion visualization on recombined images. These aspects require specific procedural planning and operator expertise.

Unlike previous narrative or critical reviews, this work is specifically designed as a practical, step-by-step guide focused on the procedural workflow and technical execution of CEM-guided biopsy in daily clinical practice.

Overall, this work is intended as a practical reference to support the implementation of CEM-guided biopsy in routine clinical practice.

## 2. Pre-Procedural Imaging Evaluation

To ensure a safe and accurate CEM-guided biopsy of breast lesions, it is mandatory to undergo at least one contrast imaging examination beforehand, preferably a CEM or, alternatively, an MRI. A careful evaluation of prior radiological images by a breast radiologist is essential to achieve optimal outcomes [14].

The principal indication for CEM-guided biopsy is a suspicious enhancing lesion detected on CEM or MRI that requires tissue diagnosis but cannot be reliably identified using conventional mammography, digital breast tomosynthesis, or targeted second-look ultrasound [9]. When the lesion is initially detected on MRI, pre-procedural CEM should confirm that the enhancement can be reproduced and that the target can be adequately included within the biopsy field.

Targeted second-look ultrasound should be performed whenever a potential sonographic correlate can be identified on the basis of lesion location and morphology. If a confident correlate is demonstrated, ultrasound-guided biopsy should generally be preferred because it provides real-time needle visualization and avoids both iodinated contrast administration and ionizing radiation. CEM-guided biopsy is therefore primarily indicated for suspicious enhancing lesions without a reliable correlate on conventional imaging [9].

The main patient-related contraindications are those that preclude the safe administration of iodinated contrast medium, as discussed in the following sections. Technical feasibility also depends on the ability to position and compress the breast adequately and to include the target within the biopsy window. Very posterior lesions, targets close to the chest wall, lesions outside the compression field, insufficient breast thickness, faint enhancement, marked background parenchymal enhancement, or rapid loss of lesion conspicuity may make targeting difficult or unfeasible [9,10,11].

If the target is not clearly visible on procedural CEM, biopsy should not be performed using estimated coordinates alone. Additional projections and careful comparison with the prior diagnostic examination should be undertaken, and low-energy mammography or digital breast tomosynthesis images should be reviewed for a corresponding mass, distortion, asymmetry, or calcifications. If a reliable morphological correlate is identified, biopsy may be performed using the most appropriate alternative guidance technique. Alcantara et al. reported one case in which reduced enhancement prevented CEM targeting, but a corresponding architectural distortion identified on digital breast tomosynthesis allowed for successful DBT-guided biopsy [9].

If the lesion remains non-visible and no reliable morphological or sonographic correlate is identified, the CEM-guided biopsy should be canceled. Non-visualization should not automatically be interpreted as evidence of benignity. The case should be reviewed according to the original degree of suspicion, and short-interval contrast-enhanced imaging follow-up or biopsy under an alternative imaging modality should be considered. In the initial CEM-guided biopsy series, lesions not visible on procedural CEM were deferred to short-term follow-up, following the approach commonly used for non-visualization during MRI-guided biopsy [9].

It is strongly discouraged to perform a biopsy without confirming the presence of a lesion or determining its precise location within the breast, as this may compromise both diagnostic accuracy and procedural success.

Pre-procedural evaluation plays a crucial role in accurately describing the target lesion and planning the biopsy, including determining the ideal patient position, selecting the shortest and safest access route through the breast tissue, choosing the appropriate needle type and trajectory, and estimating the number of tissue samples to be collected.

Ideally, it should include an analysis of previously acquired CEM images reported according to the Breast Imaging Reporting and Data System criteria (BI-RADS) and acquired in the standard cranio-caudal and medio-lateral–oblique views to measure the distances of the lesion from the nipple, skin, and other landmarks (e.g., calcifications, surgical clips, or post-biopsy markers) [15]. This step is critical for selecting the optimal approach, ensuring the shortest biopsy path to the target and thus minimizing trauma to the breast parenchyma. A significant advantage of CEM-guided biopsy over MRI-guided biopsy is the ability to match low-energy CEM views with 2D images from conventional mammography, allowing for identification of critical findings, such as calcifications, and guiding biopsy planning, which may not be revealed on pre-contrast MRI sequences [16,17].

Finally, a careful pre-procedural imaging evaluation may also identify potential biopsy contraindications and associated risks, enabling physicians to anticipate complications, particularly in superficial lesions or lesions located adjacent to the pectoral muscle.

**Figure 1:**
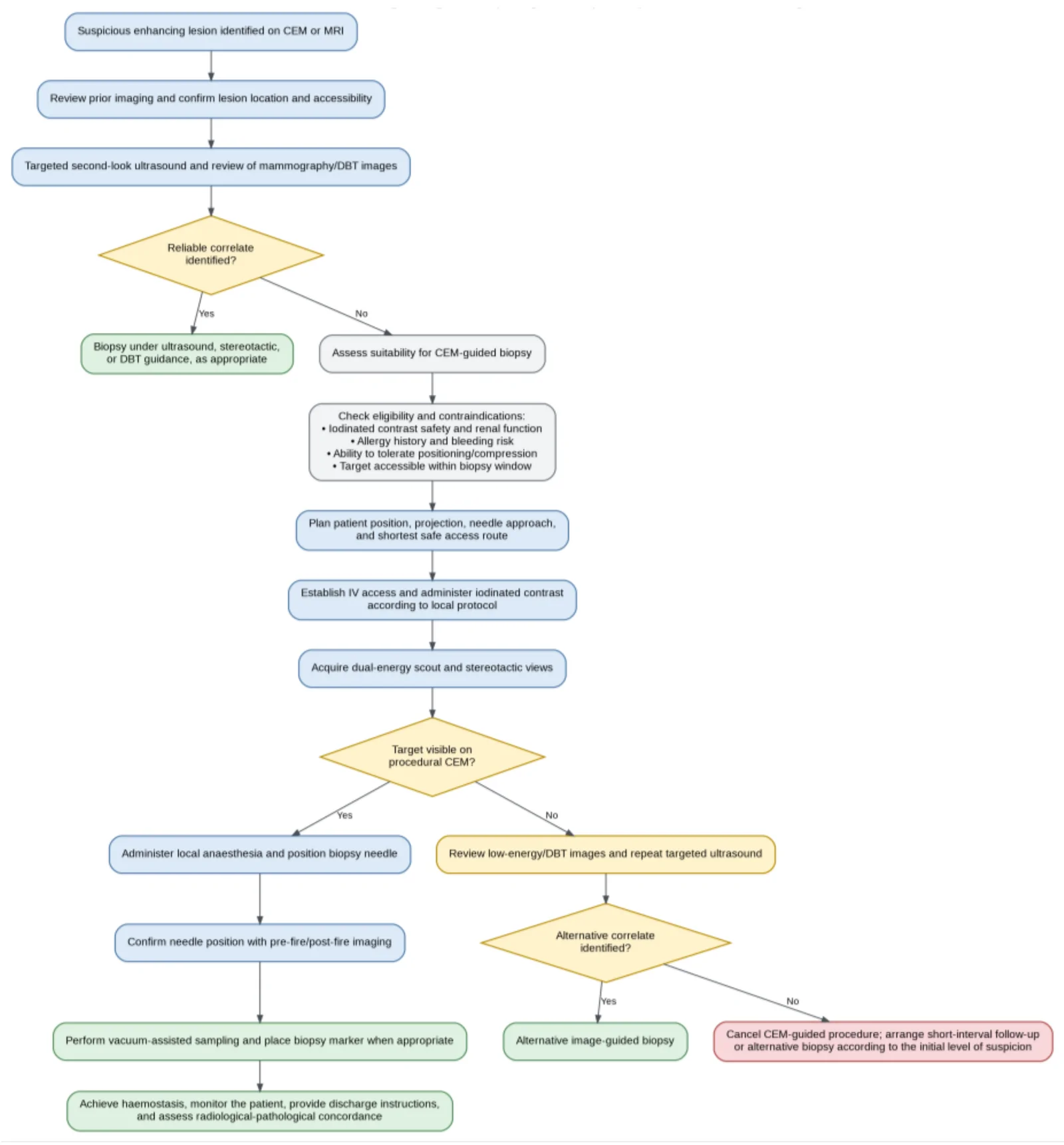
Proposed clinical and procedural workflow for CEM-guided biopsy, including patient selection, assessment for alternative biopsy guidance, procedural targeting, management of target non-visualization, tissue sampling, and post-biopsy care. CEM, contrast-enhanced mammography; DBT, digital breast tomosynthesis; IV, intravenous.

A detailed report of the pre-procedural assessment serves as an operational guide during the procedure and as a reference for the multidisciplinary board team. The complete clinical and procedural workflow for CEM-guided biopsy, from lesion assessment and patient selection to tissue sampling and post-procedural management, is summarized in Figure 1.

## 3. Patient Preparation

As with all biopsy procedures, clinical evaluation and patient preparation are critical for the success and safety of CEM-guided biopsy.

Because dedicated international guidelines for several procedural aspects of CEM-guided biopsy are not yet available, some of the practical recommendations presented below reflect our institutional practice and early published experience and may require adaptation according to local protocols, equipment, and patient characteristics.

Particular attention should be paid to obtaining informed consent, assessing the patient’s medical and pharmacological history, and evaluating laboratory parameters to assess the coagulation profile [18]. The use of ICM during the procedure also requires the evaluation of the patient’s renal function and allergy risk [18].

### 3.1. Informed Consent

Informed consent should be obtained directly by the physician performing CEM-guided biopsy, according to the national laws and institutional protocols [19]. The patient must always be informed about the indications, benefits, risks, and potential associated adverse events. If available, alternative options should also be discussed, along with a detailed explanation of the procedure, including the need for peri-procedural medications, such as anesthesia, corticosteroids, and antihistamines [14].

Meeting the patient before the procedure is the recommended practice, enabling clarification of any queries and establishing a good relationship. Written informed consent should ideally be obtained at least 24 h in advance, allowing the patient to review it, discuss it with family members, and withdraw consent if necessary. For women of childbearing age, an additional consent addendum is required to exclude the possibility of pregnancy [19].

### 3.2. Evaluation and Management of Bleeding Risk

To date, managing bleeding risk in patients undergoing CEM-guided biopsy remains a topic of debate [20,21,22]. Historically, interruption of anticoagulation or antiplatelet therapy was the standard practice. However, current evidence suggests that clinically significant hematomas post image-guided biopsies are rare, and thus routine discontinuation of such treatments is not always necessary [23].

The American College of Radiology (ACR) recommends that each case be reviewed individually [24]. The Society of Interventional Radiology (SIR) and the Cardiovascular and Interventional Radiological Society of Europe (CIRSE) agree on the importance of balancing patient-specific factors and bleeding risks during the procedure [25,26]. Although CEM-guided biopsy is classified as a low-risk procedure by CIRSE, with associated significant bleeding rates of less than 2% [25], a thorough evaluation is necessary to identify high-risk patients. A valuable tool recommended in clinical practice for assessing bleeding risk is the HEMSTOP questionnaire, based on clinical history, with demonstrated sensitivity and specificity of 89.5% and 98.6%, respectively [26,27,28,29,30]. Laboratory coagulation tests, including platelet count, prothrombin time (PT), activated partial thromboplastin time (aPTT), and fibrinogen, are generally unnecessary in patients without a history of abnormal bleeding and not on antithrombotic therapy. However, these tests are indicated in cases of positive bleeding background, ongoing anticoagulant treatment, or clinical conditions affecting the coagulation system (e.g., liver or renal disease) [26,28].

Patients on vitamin K antagonists must have a platelet count above 20 × 10^9^/L and an INR below 2.0. For patients on anticoagulation therapy without a high thromboembolic risk, the recommendations from the CIRSE Consensus Guidelines (version 6) may be followed. Conversely, in patients at high risk of thromboembolism, it is advisable to continue anticoagulation therapy with careful monitoring [26].

### 3.3. Evaluation and Management of ICM Allergic Risk

According to the 2020 SIRM-SIAAIC consensus document, a detailed medical history should be obtained before ICM administration to identify patients at risk [31]. The main risk factors include previous allergic or allergic-like reactions to the same class of ICM, uncontrolled asthma, urticaria or angioedema, mastocytosis, and idiopathic anaphylaxis; allergies to seafood, other foods, or unrelated drug classes are not considered risk factors [32,33]. ESUR and SIRM-SIAARTI recommendations advise against routine premedication, including in at-risk patients, because of insufficient evidence of efficacy and the possibility of masking early signs of a reaction [34,35,36]. In patients with a previous hypersensitivity reaction, an alternative imaging method or a different contrast agent should be considered. When alternatives are not feasible, allergist consultation and skin testing may help identify a lower-risk, non-cross-reactive agent [37,38,39,40,41,42,43,44]. Patients with a positive allergic history require close monitoring of vital signs and immediate readiness to manage potential emergencies.

### 3.4. Evaluation and Management of Contrast-Induced Acute Kidney Injury

Post-contrast acute kidney injury (PC-AKI) is defined as an increase in serum creatinine of ≥0.3 mg/dL or ≥1.5 times the baseline value within 48–72 h after ICM administration, or urine output <0.5 mL/kg/h for at least 6 h [34]. Pre-existing renal impairment is the main recognized risk factor, although ICM dose and osmolarity may also contribute [34]. Renal function should be assessed using eGFR calculated with the CKD-EPI equation rather than serum creatinine alone [45]. An eGFR <30 mL/min/1.73 m^2^ is considered the principal risk threshold for intravenous ICM administration; eGFR should be measured within 7 days in hospitalized patients or those with unstable renal function and within 3 months in clinically stable patients [46]. In patients at risk, low- or iso-osmolar contrast agents should be used at the lowest diagnostically adequate dose [47,48,49]. Preventive intravenous hydration with sodium bicarbonate or saline, followed by reassessment of eGFR 48 h after the procedure, is recommended according to current guidelines [46].

### 3.5. Peri-Procedural Drug Administration

A well-calibrated peripheral venous access (21–22 Gauge) should be established before the ICM injection to ensure immediate drug administration in case of an emergency [50]. Peripheral intravenous access is generally placed in the basilic vein of the arm. No evidence suggests a preference for the ipsilateral arm to the lesion to be biopsied or for the opposite arm in patients undergoing axillary lymphadenectomy.

Preparatory fasting is not required before routine intravascular administration of modern non-ionic iodinated contrast media, as there is no evidence that fasting reduces the risk of nausea, vomiting, or aspiration [34]. Accordingly, fasting is not routinely required before CEM-guided biopsy performed under local anesthesia. When sedation or general anesthesia is planned, fasting should follow the applicable anesthetic and institutional protocols.

Routine antibiotic prophylaxis is not recommended, as infection rates are low when sterile technique is used during the procedure. However, prophylactic antibiotics may be considered in immunocompromised patients. No specific antibiotic type is indicated, and there is no evidence to suggest the superiority of prolonged (three-day) therapy over shorter courses (one-day) [51].

### 3.6. Patient Position

The patient may be positioned in the prone (PP), upright (orthostatic, UP), or lateral decubitus (LP) position, with the breast compressed by a fenestrated plate [13].

**Figure 2:**
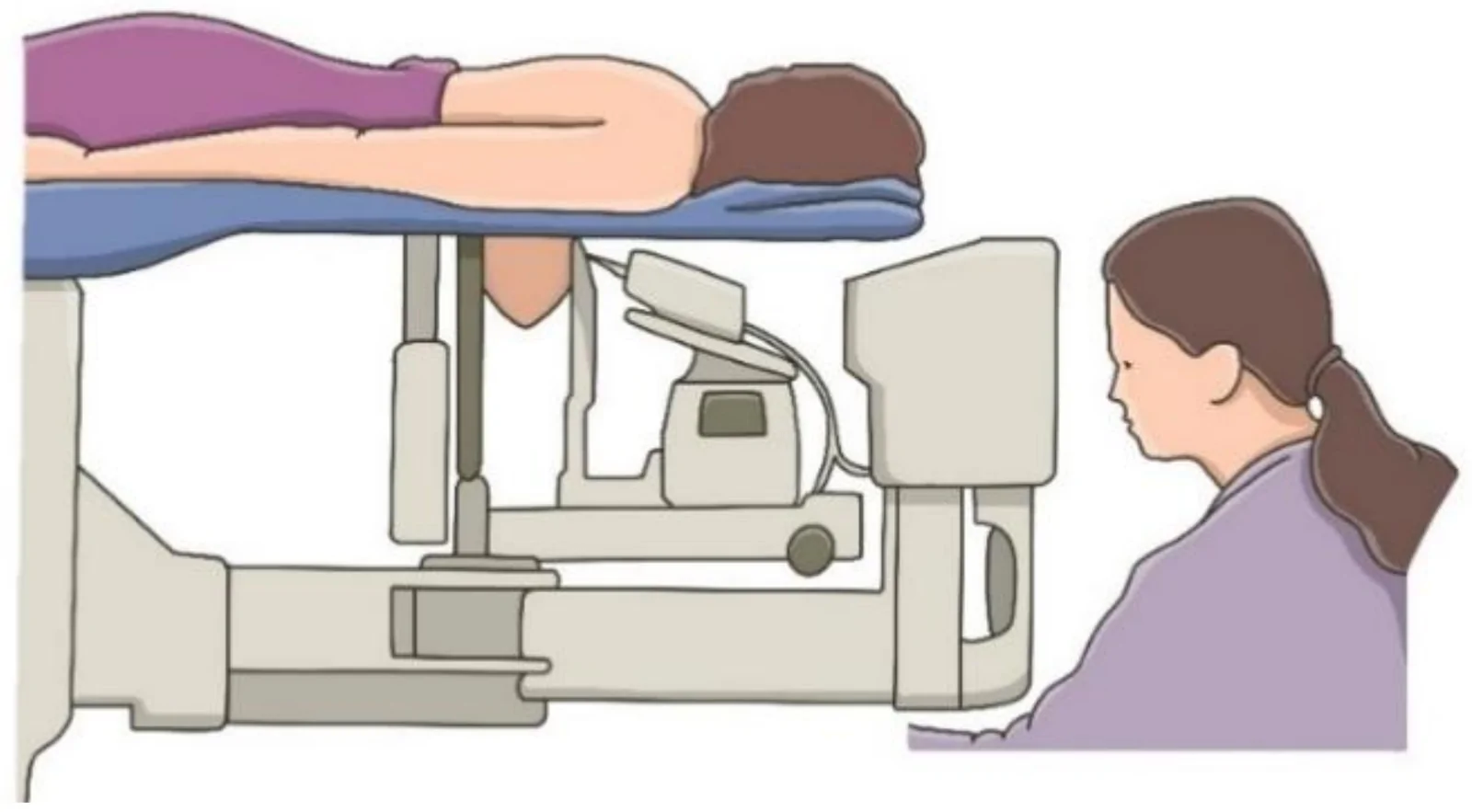
The patient lies prone, with the affected breast placed through a table opening and compressed against the detector by a fenestrated plate for biopsy.

In the PP, the patient lies face down on the table; the affected breast is suspended through an opening and compressed against the detector under the table by a plate equipped with a window, through which the radiologist performs the biopsy. This design ensures that only the affected breast is exposed and aligned with the mammographic unit and the biopsy equipment (Figure 2). The PP reduces stress reactions and minimizes patient movement, making it preferable to other positions. Studies indicate a fainting risk of 0% compared to UP approaches, where vasovagal reactions occur in 3.2% of cases. It also reduces the likelihood of procedure interruptions due to patient discomfort or requests [12,13]. However, the PP may not be suitable for patients with respiratory failure or severe obesity, as they may experience breathing difficulties. Careful assessment is required in these cases to determine the optimal biopsy site.

In the LP, the patient lies on their side with the breast compressed laterally, while in the UP, the patient sits on a chair with compression applied cranio-caudally or laterally. These approaches provide a close view of the operative field and biopsy needle, which, although beneficial for accuracy, may increase patient discomfort, anxiety, and the risk of fainting, resulting in involuntary movements and procedural interruptions [52].

## 4. Technical Specifications

### 4.1. ICM Administration and Imaging-Guided Target Lesion Localization

After positioning the patient correctly, the breast is compressed, and a standard mammogram view is obtained to verify correct target centering within the biopsy window, based on distances or landmarks identified during the pre-procedural planning phase [53]. It is advisable to acquire an initial view, perhaps integrated with tomosynthesis, at this stage: the reduction in tissue overlap and the masking effect caused by tissue density should help avoid potentially risky anatomical structures, particularly large-caliber vessels, which are at risk of hematoma [54].

Additionally, it is preferable to outline the margins of the target area on the skin through the biopsy window using an indelible skin marker to maintain accurate localization, as the breast will need to be decompressed during ICM administration to ensure better flow into the mammary vessels [12,13].

According to our institution’s guidelines for CEM examinations, the protocol involves the intravenous administration of ICM (Iopamiro 370 mg I/mL) at a dose of 1.5 mL/kg, injected at a rate of 3 mL/s. Approximately two minutes after the end of the injection, the breast is recompressed, and a dual-energy scout view (0 degrees) is acquired to visualize the target-enhancing lesion on the recombined image. Because targeting depends on lesion enhancement, accurate timing between contrast administration and image acquisition is essential. Subsequently, two additional dual-energy views are obtained at ±15° from the perpendicular, with the target positioned at the center of the compression plate opening. The radiologist localizes and identifies the lesion, while the software calculates the X, Y, and Z coordinates from the images to guide the needle placement. The exact contrast agent, injection rate, decompression and recompression sequence, and acquisition timing may vary according to the mammography system, manufacturer recommendations, and institutional workflow.

Radiation exposure should be considered during CEM-guided biopsy because each procedural image consists of a dual-energy acquisition, and the cumulative average glandular dose (AGD) increases with the number of scout, pre-fire, post-fire, and post-biopsy acquisitions. In one initial single-center series, the mean total procedural AGD was 14.3 ± 12.3 mGy. This value was influenced by one patient with previous liquid-silicone breast augmentation; after exclusion of this patient, the mean AGD was 10.7 ± 2.5 mGy. This was comparable to the reported dose for digital breast tomosynthesis-guided biopsy (10.18 ± 3.73 mGy) and lower than that reported for conventional stereotactic biopsy (22.06 ± 14.92 mGy) [11]. The AGD per individual exposure remained below 3 mGy in that study [11]. However, these findings are based on a small cohort and should not be considered universal reference values, because procedural dose may vary according to breast thickness and composition, equipment, acquisition parameters, and the number of repeated images.

Dose optimization should follow the as-low-as-reasonably-achievable principle. Careful review of previous examinations, selection of the optimal projection and needle approach, and pre-procedural localization of the target relative to the skin and anatomical landmarks may facilitate inclusion of the lesion within the biopsy window on the first scout acquisition [11]. Unnecessary repeat scout and stereotactic acquisitions should be avoided, while additional images should still be obtained whenever required for safe and accurate needle positioning and sampling. Efficient procedural workflow may also reduce repeated acquisitions caused by progressive loss of lesion conspicuity. Because cumulative dose depends on the number of dual-energy acquisitions, the number of images and total procedural AGD should be monitored as part of local dose optimization practice.

### 4.2. Biopsy Technique

Once the access site has been identified and the skin sterilized, the radiologist anesthetizes the skin and deeper breast tissue at the coordinates [55,56]. The depth of anesthesia administration must account for the entire length of the sampling port and the biopsy needle tip, rather than stopping at the Z coordinate (the center of the sampling port) [53]. At our institution, approximately 10–20 mL of 1–2% lidocaine is generally used, although the required volume and concentration should be adapted to lesion depth, access route, patient characteristics, and local practice. In patients without cardiac disease, lidocaine may be combined with epinephrine to reduce the bleeding risk, taking advantage of its vasoconstrictive effect. The maximum recommended dose of 1% lidocaine with epinephrine is 7 mg/kg, not exceeding 500 mg, which corresponds to 50 mL in a 70 kg patient [57].

For superficial targets near the skin surface, a useful technical maneuver employed at our institution is to inject the local anesthetic into the space between the skin and the lesion, creating a “bombé effect” that displaces the target deeper and may reduce the risk of skin injury during vacuum-assisted sampling.

A subsequent dual-energy pre-fire acquisition at 15 degrees is advised to assess any target displacement due to the anesthetic injection and to confirm the needle tip is positioned at the edge of the target. The radiologist inserts the needle to the calculated depth and reviews post-fire views to ensure that the needle’s sampling window is within the lesion [53].

These procedures in CEM-guided biopsy are not always successful, particularly in cases of faint or rapidly fading enhancement. In this context, CEM-guided biopsy differs from standard stereotactic techniques, as lesion visibility is dependent on contrast enhancement and may decrease over time. This requires efficient workflow management and rapid execution of targeting and sampling steps to avoid loss of lesion conspicuity.

**Figure 3:**
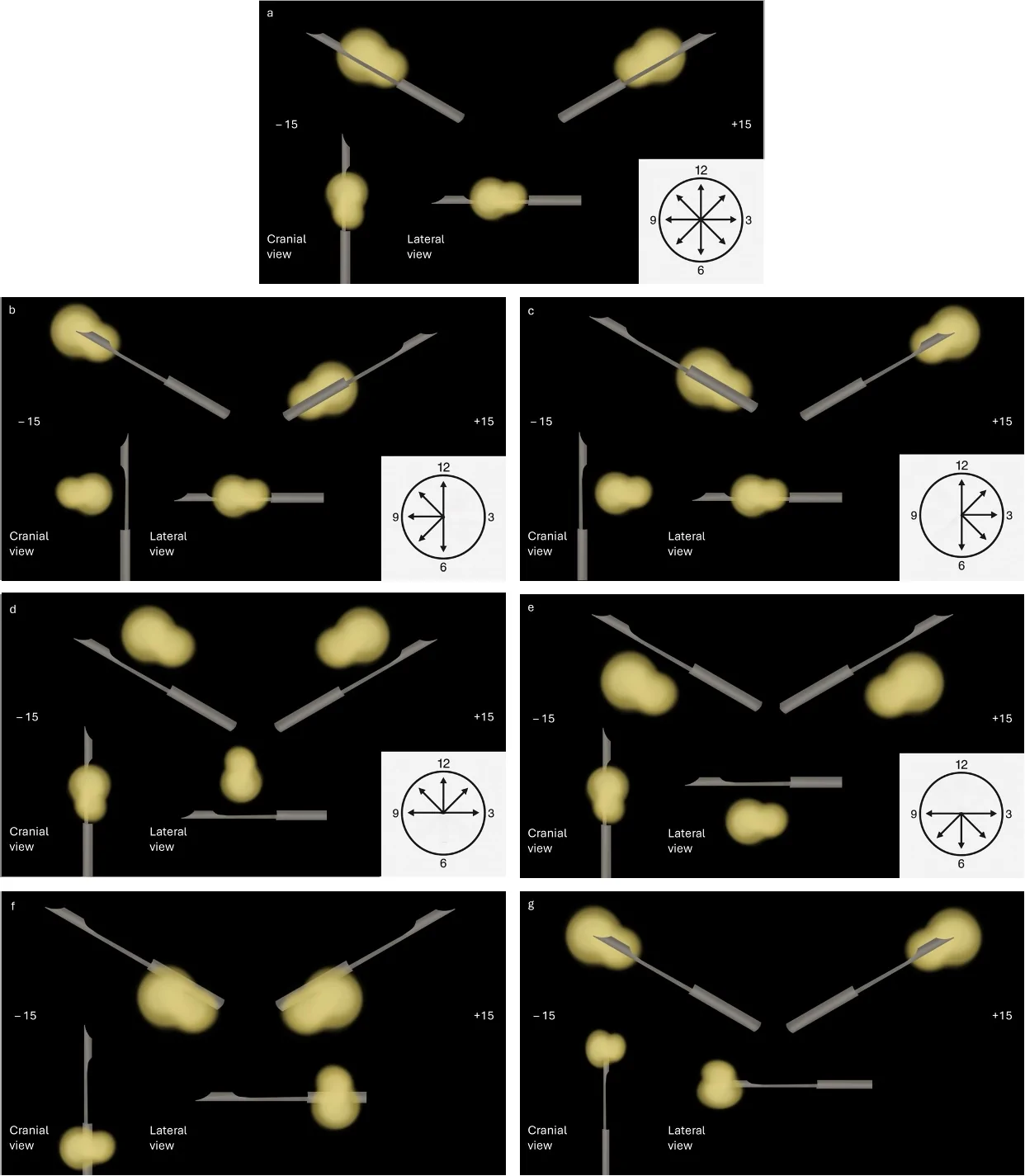
(**a**) A diagram illustrating the optimal positioning for CEM-guided biopsy. Two post-fire recombined images show the needle cavity centered within the breast lesion. Since the needle is perfectly aligned with the target, tissue samples can be collected 360° around the cavity. (**b**,**c**) Two examples of horizontal misalignment: in (**b**), the needle is positioned to the right of the target, in (**c**) to the left. Sampling should be performed to the left (**b**) and right (**c**) of the needle, respectively. (**d**,**e**) Two examples of vertical misalignment: in (**d**) the needle lies below the target, in (**e**) above it. Sampling should therefore be performed above (**d**) and below (**e**) the needle, respectively. (**f**,**g**) Two examples of depth misalignment: in (**f**), the needle is too deep compared to the target, in (**g**) it is too superficial. The needle should be retracted and imaging repeated to ensure the target is within the cavity (**f**), or the needle should be advanced, and imaging repeated to confirm correct positioning (**g**).

After needle placement, it may be incorrectly positioned, too far left or right, above or below, or either too deep or too shallow relative to the target. Figure 3 illustrates how to redirect or adjust the needle position in case of such errors.

Sampling is typically performed using a vacuum-assisted biopsy device (VABB), a dedicated system that creates a vacuum inside the needle, enabling tissue samples to be obtained via aspiration [58,59].

Once the biopsy is completed, the radiologist inserts a marker clip into the biopsy cavity and withdraws the needle. A final dual-energy image is acquired to confirm the clip’s correct placement. Afterwards, the needle is removed, and the patient is released from compression.

CEM-guided biopsy requires familiarity with both stereotactic or DBT-guided breast intervention and the specific technical characteristics of contrast-enhanced imaging, including contrast timing, lesion visibility on recombined images, patient positioning, and rapid targeting before enhancement decreases. Operator experience may therefore influence procedural efficiency, the number of repeated acquisitions, radiation exposure, and the likelihood of successful lesion targeting, particularly for faintly enhancing, superficial, or difficult-to-access lesions [9,10,11,12,13]. However, the learning curve for CEM-guided biopsy has not yet been formally quantified. Initial implementation should include dedicated system-specific training, review of procedural protocols, and, when feasible, supervision by radiologists experienced in image-guided breast biopsy. Future studies should evaluate procedural success, procedure duration, and radiation exposure according to operator experience.

## 5. Post-Biopsy Assessment

Providing the patient with all the necessary wound care instructions is a fundamental part of the procedure. After the biopsy, hemostasis should be achieved by direct manual compression, followed by application of a compressive dressing, with or without an ice pack [60,61]. The duration of compression should be individualized and continued until adequate hemostasis is achieved. Patients should then be observed for approximately 15–60 min, depending on the procedure and the patient’s clinical profile [60]. Before discharge, patients should receive appropriate instructions regarding wound care and possible delayed complications, including pain, bleeding, swelling, and hematoma [60,62].

Pain management is a critical part of the recovery phase. Without specific allergies or liver disease, the patient may take paracetamol every 6–8 h, up to a maximum of 3 g per day. Aspirin and other non-steroidal anti-inflammatory drugs (NSAIDs) should be avoided due to the increased risk of bleeding [63].

## 6. Main Complications and Management

It is essential to anticipate all possible complications and plan for their timely recognition and appropriate treatment. This requires rapid access to drugs, resuscitation equipment, imaging techniques, and interventional radiology or surgical procedures [64].

Below, we identify and discuss the management of the two main complications—bleeding and acute adverse reactions—exploring interventional strategies and the most up-to-date guidelines for effective and safe treatment in patients undergoing CEM-guided biopsy.

### 6.1. Bleeding

Severe bleeding (grade 3 or higher) is a rare complication of CEM-guided biopsy. In a retrospective monocentric study of 37 CEM-guided biopsy cases, Nori et al. reported a post-procedural bleeding rate of 27%, with only one severe case (2.7%) requiring surgical curettage, resulting in no long-term consequences [12]. This is consistent with other standard VABB procedures, in which the overall hematoma rate is approximately 30% and severe bleeding occurs in 2% of cases [13,65]. These results indicate that CEM-guided biopsy is safe and acceptable in terms of procedural bleeding risks.

Severe bleeding may be diagnosed during or immediately after the procedure using imaging techniques, mainly US, or through clinical symptoms such as pain, tachycardia, and hypotension [66].

The clinical severity and bleeding site should be identified immediately, and manual compression with ice application should be performed. Depending on the severity, determined clinically and through imaging, the following treatment options may also be considered [67]:

- Compression and local ice application for small hematomas;
- Observation and US follow-up for small hematomas, which often resolve spontaneously within 2–3 weeks;
- Antidotes for any anticoagulant drugs taken by the patient;
- Surgical consultation is rare and reserved for large or complicated hematomas that do not respond to conservative treatment.

### 6.2. Acute Adverse Reactions

Acute adverse reactions to ICM usually occur within one hour of administration and include allergic and pseudoallergic reactions [34,68]. They are classified as mild, moderate, or severe according to clinical severity. Allergic or pseudoallergic reactions occur in approximately 0.05–0.1% of patients undergoing ICM imaging [33]. Mild reactions are generally self-limiting, moderate reactions may require pharmacological treatment, and severe reactions constitute medical emergencies requiring immediate intervention [69]. Severe acute reactions occur in approximately 0.01–0.2% of cases and usually develop within the first few minutes after ICM administration [39,68]. The main symptoms, severity categories, and management are summarized in Table 2.

## 7. Conclusions

CEM-guided biopsy is a promising technique for the percutaneous diagnosis of enhancing breast lesions and may offer practical advantages over MRI-guided biopsy, including shorter procedure times, greater accessibility, and improved patient tolerability. However, the available evidence is still limited and is largely derived from retrospective, single-center studies and relatively small patient cohorts. Consequently, the reported technical success rates, procedural efficiency, and safety outcomes should be interpreted cautiously, and the current evidence does not yet establish the superiority of CEM-guided biopsy over other image-guided biopsy techniques. Larger prospective and multicenter studies using standardized protocols are required to define its clinical role more clearly.

Furthermore, direct quantitative comparisons with other biopsy guidance techniques, particularly MRI-guided biopsy, are still limited.

Additionally, the management of complications and risk assessment must be continually updated based on emerging scientific evidence and clinical experience.

This practical guide serves as a valuable reference for clinicians new to CEM-guided biopsy, outlining the key technical and clinical aspects of the procedure, from pre-procedural assessment to management of the main complications. The aim is to provide a detailed overview of the method and offer practical insights to help overcome operational challenges, making the technique more easily applicable in routine clinical practice. Moreover, the technique presents specific technical challenges related to contrast dynamics and lesion visualization, which distinguish it from other image-guided biopsy methods and require dedicated training. We hope that this guide will serve as a helpful tool for physicians, facilitating the wider adoption and standardization of the procedure.

## Figures and Tables

**Table 1:** Summary of main published clinical studies on CEM-guided biopsy: Technical Success, Diagnostic Outcomes, Procedure Time, Radiation Exposure, and Complications.

Study	Year	Study Design	Patients/Lesions	Technical Success	Diagnostic Accuracy/Concordance	Malignancy or Histological Yield	Procedure Time	Radiation Exposure	Complications
Alcantara et al. [9]	2023	Retrospective review of a prospectively enrolled feasibility cohort	64 patients/66 lesions	95.4% (63/66)	Formal diagnostic accuracy NR	39.7% malignant (25/63 successful biopsies)	Median 15 min (IQR 4; range 9–25; *n* = 59)	NR	Early hematoma 22.2% (14/63); late hematoma 4.8% (3/63); vasovagal reaction 3.2% (2/63); no severe complications or allergic reactions
Kornecki et al. [10]	2023	Prospective single-center trial	50 women/51 lesions	90.2% (46/51); cancelation rate 9.8% (5/51)	Pathology concordant in all 46 biopsied lesions; formal diagnostic accuracy NR	23.9% malignant (11/46); 21.7% high-risk (10/46); 54.3% benign (25/46)	Mean 16.6 min; median 15.4 min	NR	NR
Tang and Cheung [11]	2023	Retrospective single-center initial experience	12 women/12 lesions	100% (12/12)	Formal diagnostic accuracy NR	66.7% malignant (8/12)	Mean 17 ± 6.3 min (range 6–27)	Mean AGD 14.3 ± 12.3 mGy	No major complications, including allergic reactions, vasovagal reactions, or infections
Aribal et al. [4]	2024	Retrospective single-center analysis of MRI-only detected lesions	26 women/29 referred lesions; 27 lesions biopsied	93.1% (27/29)	Biopsy PPV 33.3%; formal diagnostic accuracy NR	33.3% malignant (9/27): 5 invasive cancers and 4 DCIS	Median 14 min; 24 min shorter than MRI-guided biopsy at the same institution	NR	Minor complications 25.9% (7/27), mainly small hematomas; vasovagal reaction 3.7% (1/27)
Nori Cucchiari et al. [12]	2024	Retrospective single-center analysis using a prone CEM-guided biopsy system	37 enhancing-only lesions	100% (37/37)	Formal diagnostic accuracy NR	Significant histological finding rate 97.3%; malignancy rate NR	Mean 15.8 min	Dose distribution evaluated, but exact dose NR in the abstract	Complications in 27% of cases, primarily hematomas; one severe case (2.7%) required surgical curettage

Abbreviations: AGD, average glandular dose; CEM, contrast-enhanced mammography; DCIS, ductal carcinoma in situ; IQR, interquartile range; MRI, magnetic resonance imaging; NR, not reported; PPV, positive predictive value.

**Table 2:** Acute adverse reactions to ICM.

**Mild** Symptoms are generally self-limiting with no evidence of progression and should be monitored	
**Allergic/allergy-like**	**Management**
- Localized wheals/mild pruritus - Localized cutaneous edema - Mild throat discomfort/tickling sensation - Nasal congestion - Sneezing/conjunctivitis/rhinorrhea	No treatment required; clinical monitoring only
**Moderate** Symptoms are more noticeable, and some can become serious if left untreated	
**Allergic/allergy-like**	**Management**
- Diffuse wheals/intense pruritus - Diffuse erythema - Facial edema without dyspnea - Sensation of throat tightness/hoarseness - Mild bronchospasm/shortness of breath without hypoxia	Chlorpheniramine 10 mg IM/IV Chlorpheniramine 20 mg IM/IV Chlorpheniramine 20 mg IM/IV β2-agonists (Salbutamol) with a metered dose inhaler, 2 sprays up to three times
**Severe** Symptoms are often life-threatening. They can worsen to pulmonary edema or cardiorespiratory arrest	
**Allergic/allergy-like**	**Management**
- Dyspnea - Diffuse erythema with mucocutaneous involvement - Laryngeal edema with stridor/hypoxia - Severe bronchospasm - Significant hypoxia (absolute or relative, worsening, SpO_2_ that does not improve with oxygen administration) - Anaphylactic shock (severe hypotension, brady-tach-arrhythmias)	*Laryngeal edema*
	Epinephrine 0.5 mg IM, repeatable up to 1 mg + oxygen therapy (6–10 L/min)
	*Bronchospasm/hypoxia*
	β2-agonists (Salbutamol) + Epinephrine 0.5 mg IM, repeatable up to 1 mg
	*Anaphylaxis*
	- Call the resuscitation team - Secure the airway if necessary - Elevate the patient’s lower limbs - Oxygen therapy (6–10 L/min) - Epinephrine IM (same dose as for laryngeal edema) - IV fluids (normal saline or Ringer’s lactate, up to 2 L) - H1 antihistamines (e.g., diphenhydramine 25–50 mg IV)

IM: intramuscular; IV: intravenous.

## Data Availability

No new data were created or analyzed in this study. Data sharing is not applicable to this article.
